# Hyperoside as a Potential Natural Product Targeting Oxidative Stress in Liver Diseases

**DOI:** 10.3390/antiox11081437

**Published:** 2022-07-25

**Authors:** Eungyeong Jang

**Affiliations:** 1 Department of Internal Medicine, College of Korean Medicine, Kyung Hee University, 26, Kyungheedae-ro, Dongdaemun-gu, Seoul 02447, Korea; obliviona79@naver.com or 044409@khu.ac.kr; 2 Department of Internal Medicine, Kyung Hee University Korean Medicine Hospital, 23, Kyungheedae-ro, Dongdaemun-gu, Seoul 02447, Korea

**Keywords:** hyperoside, pharmacological effects, liver disease, antioxidant

## Abstract

Hyperoside (Hyp), also known as quercetin-3-*O*-galactoside or 3-*O*-β-D-galactopyranosyl, is a well-known flavonol glycoside that is abundant in various fruits, vegetables, and medicinal plants. Hyp has been suggested to exhibit a wide range of biological actions, including cardiovascular, renal, neuroprotective, antifungal, antifibrotic, and anticancer effects. Accumulating evidence supports the pharmacological activities of Hyp in improving liver pathophysiology. Hence, the present literature review aims to summarize preclinical data suggesting the beneficial effects and underlying mechanisms of Hyp. In addition, our study focuses on hepatic antioxidant defense signaling to assess the underlying mechanisms of the biological actions of Hyp that are closely associated with liver diseases. Experimental findings from an up-to-date search showed that Hyp possesses hepatoprotective, antiviral, antisteatotic, anti-inflammatory, antifibrotic, and anticancer activities in cellular and animal models related to liver dysfunction by enhancing antioxidant responses. In particular, hepatocellular antioxidant defense via activation of erythroid-related nuclear factor 2 by Hyp chiefly explains how this compound acts as a therapeutic agent in liver diseases. Thus, this review emphasizes the therapeutic potential of Hyp as a strong antioxidative substance that plays a crucial role in the regulation of various liver disorders during their pathogenesis.

## 1. Introduction

Liver diseases, characterized by various liver injuries from steatosis to cancer, have steadily increased the global burden as one of the leading causes of morbidity and mortality worldwide. The prevalence of chronic liver disease (CLD) is currently estimated to reach 1.5 billion across the world, and CLD ranks fourth among diseases causing death in middle-aged adults (45–64 years) [1,2]. Common risk factors that can develop and aggravate various liver diseases include viral infection, heavy alcohol use, toxin exposure, drug overuse, overnutrition, and autoimmune responses. 

Among them, excessive alcohol intake, viral hepatitis, and obesity-related metabolic disorders are three crucial risk factors, accounting for over 90% of patients with CLD [3]. While viral infection has been commonly regarded as the main etiology of CLD in the past, current issues in hepatology have frequently focused on nonalcoholic fatty liver disease (NAFLD) and alcoholic liver disease (ALD) [4,5]. 

Several signaling pathways can be intricately intertwined among pathophysiological mechanisms that cause liver diseases, and oxidative stress induced by reactive oxygen species (ROS) is considered to be one of the main pathways associated with various liver disorders [6]. In particular, the liver is vulnerable to ROS attack, and it is also known as a crucial regulator that plays key roles in the development of liver diseases with high prevalence, including NAFLD, ALD, and drug-induced liver injury (DILI) [7,8,9].

Thus, some antioxidants, such as vitamin C/E and *N*-acetylcysteine (NAC), have been used to control oxidative-stress-induced liver dysfunction. However, evidence is yet to confirm the efficacy and safety of antioxidants as curative therapies to prevent and treat liver diseases [9,10]. Considering the potential of antioxidative strategies for the treatment of liver diseases, natural antioxidants, to some extent, can be rational candidates with the possibility of beneficial actions. Hyperoside (Hyp, quercetin-3-*O*-galactoside), a flavonol glycoside, can serve as a promising antioxidant to control hepatic oxidation in liver diseases. 

Hyp has been known to be derived from many herbal plants, including *Crataegus pinnatifida*, *Abelmoschus manihot*, *Hypericum perforatum*, *Geranium carolinianum*, *Zanthoxylum bungeanum*, the flowers of *Acacia melanoxylon*, *Rosaceae*, *Rhododendraceae*, *Werspearaceae*, and *Leguminosae* [11,12,13,14]. As a potential medicinal constituent, Hyp has been reported to possess a wide range of pharmacological activities against lung cancer, cardiac injury, renal injury, lung fibrosis, and diabetes [15,16,17].

Notably, recent studies have shown that Hyp can act as an effective agent that can ameliorate various liver injuries triggered by toxin exposure, drug overuse, viral infection, and a high-fat diet. However, to the best of our knowledge, previous studies have not reviewed the pharmacological activities of Hyp in diverse liver diseases. Moreover, the association between antioxidant defenses in the liver and the central mechanisms involved in the biological actions of Hyp in treating liver diseases has not yet been identified. Therefore, the present study reviews the recent experimental results on the beneficial role of Hyp and the underlying molecular mechanism by which it regulates various conditions related to liver diseases to provide preclinical evidence for further well-designed studies that can be applied in clinical settings and drug development.

## 2. Methods

A comprehensive search for relevant preclinical studies on Hyp and pharmacological effects related to liver diseases published from inception to May 2022 was performed using PubMed (http://pubmed.ncbi.nlm.nih.gov/, accessed on 1 June 2022), EMBASE (https://www.embase.com/, accessed on 1 June 2022), CNKI (https://oversea-cnki-net-ssl.openlink.khu.ac.kr/index/, accessed on 1 June 2022), and Google Scholar (http://scholar.google.com/, accessed on 1 June 2022). The following key search terms were entered: (“hyperoside” OR “hyperin” OR “quercetin-3-O-galactoside” OR “3-O-β-D-galactopyranosyl”) AND (“liver” OR “hepato” OR “hepatic”) NOT (“human” OR “clinical”). To retrieve as many related articles as possible, all studies obtained from reference lists as well as the above two databases were manually reviewed. After excluding duplicated or inapplicable studies, 35 articles were reviewed meticulously in this study. 

## 3. Phytochemistry of Hyperoside

**Figure 1:**
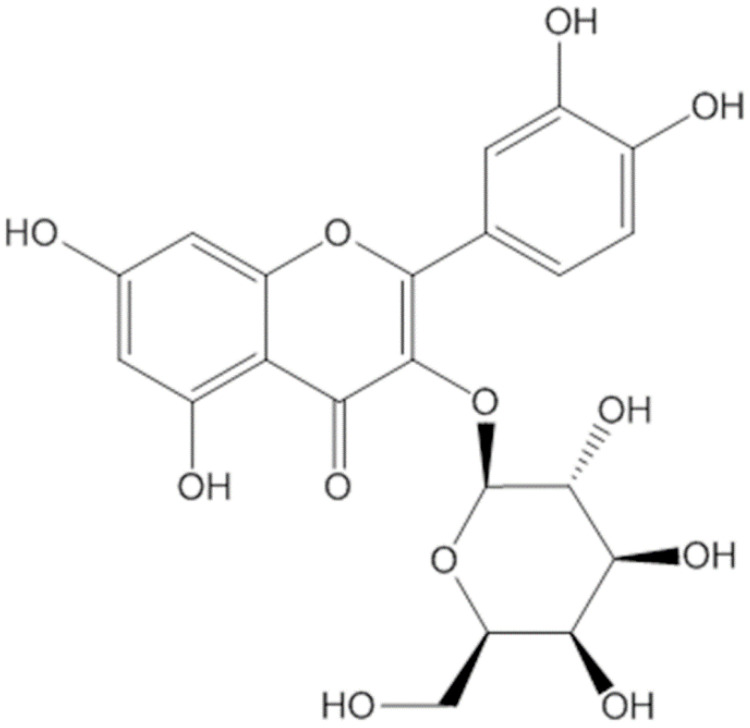
The chemical structure of Hyp.

Hyp (PubChem CID: 5281643), a synonym for 3-*O*-galactoside of quercetin, is a well-known antioxidant flavonoid frequently found in many herbal plants. The formal name is 2-(3,4-dihydroxyphenyl)-3-(β-D-galactopyranosyloxy)-5,7-dihydroxy-4H-1-benzopyran-4-one, and its molecular formula is C_21_H_20_O_12_ with a molecular weight of 464.3793 g/mol (Figure 1). Hyp is a type of flavonoid possessing many biological functions and a secondary metabolite serving a role as valuable natural products [18]. It is reported that the genera *Hypericum*, *Arbutus*, *Abelmoschus*, *Zanthoxylum*, *Houttuynia*, etc. are rich in Hyp, and it has been extracted from over 30 herbal plants [14,19,20,21,22,23,24]. 

For instance, high-performance liquid chromatography (HPLC) chromatography for detecting Hyp contents showed the quantification of Hyp at 353 nm of UV and 16.13 min of retention time. The leaf parts of the plant showed larger amount of Hyp than the parts of the flower and stem. In addition, the content of Hyp in *Hypericum perforatum* was superior over that in *Hypericum leptophyllum* [24]. Similarly, 96% ethanol extract of *Hypericum perforatum* showed higher contents of Hyp compared with rutin and quercetin [23]. 

The isolation of Hyp from medicinal plant extracts mostly used ethanol solvent and the extraction methods of reflux and ultra-sonication with a range of purity from 1.91–91.41% [14]. As depicted in Figure 1, the molecular structure of Hyp is composed of multiple polar groups that include one carbonyl group, three ether linkages, and eight hydroxyl groups [25]. 

A recent study reported that Hyp contents obtained from *Hypericum* species can be analyzed quantitatively and qualitatively using high-performance thin-layer chromatography (HPTLC) methods [26]. However, the intact detection of Hyp using high-performance liquid chromatography-ultraviolet (HPLC-UV) or liquid chromatography-tandem mass spectrometry (LC-MS-MS) methods is nearly impossible because of the galactose sugar moiety of Hyp. Instead, the use of HPLC-UV analysis to detect quercetin following enzymatic hydrolysis with β-glucuronidase and sulfatase is able to show the pharmacokinetic profiles of Hyp, including t_max_ and C_max_ [27]. 

However, compared to isoquercitrin (quercetin-3-*O*-glucoside), the absorption rate in the gastrointestinal tract and hydrolyzation of Hyp were poorer owing to the type of sugar moiety [28]. Thus, there are some limitations to the clinical application of Hyp, owing to its poor solubility, low bioavailability, and instability. Therefore, several novel tools, such as nanotechnology, microencapsulation, and eutectic mixtures, have been investigated to enhance the bioavailability and clinical potential of Hyp [29]. Moreover, further researches on the optimized extraction method, structure-based analysis, and technology improving yield of Hyp need to be conducted for clinical use. 

## 4. Pharmacological Effects of Hyperoside in Liver Diseases

### 4.1. Hepatoprotective Effects

The liver is involved in various physiological functions, including the metabolism, immune responses, excretion, and detoxification [30]. Many factors, such as overnutrition, ethanol, drugs, and xenobiotics, lead to dysregulation of liver function. Hepatic injury is a major health problem worldwide [31]. Usually, liver injury leads to pathological manifestations in which abnormal serum markers and histological deterioration are observed due to hepatocyte death and the activation of hepatic stellate cells (HSCs) and Kupffer cells [31]. Considering that liver injury has been chiefly implicated in triggering various liver disorders, the beneficial roles of hepatoprotective agents have been emphasized for the treatment or prevention of liver and biliary tract diseases [32,33].

When rodents or liver cells are exposed to toxic chemicals, such as carbon tetrachloride (CCl_4_), hydrogen peroxide (H_2_O_2_), tert-butyl hydroperoxide (t-BHP), alcohol, and D-galactosamine (d-GalN), significant alterations in their liver function are observed, with substantial evidence that elevated serum liver enzymes correlate with the severity of liver injury. These high levels of serum aspartate aminotransferase (AST) and alanine aminotransferase (ALT) are mostly accompanied by histological deterioration of the liver tissue [34,35,36,37,38,39].

**Figure 2:**
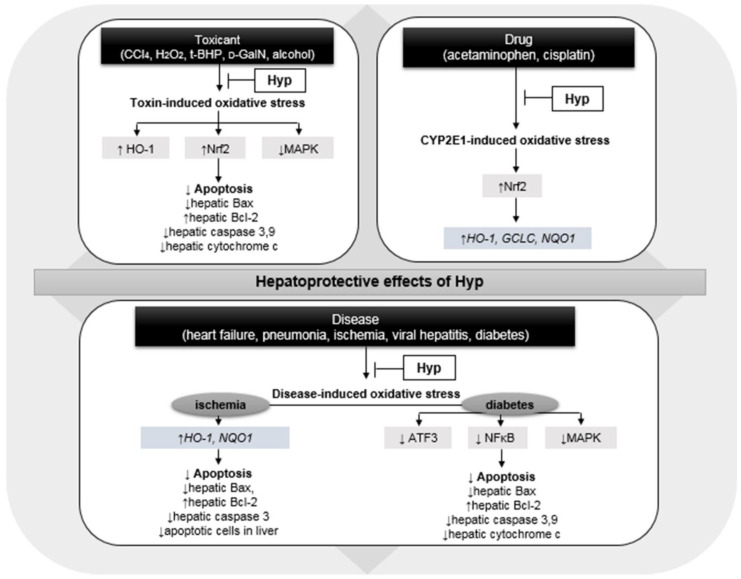
Hepatoprotective effects of hyperoside (Hyp) via the regulation of oxidative stress triggered by toxic chemical, drug, and various diseases; CCl_4_, carbon tetrachloride; H_2_O_2_, hydrogen peroxide; t-BHP, tert-butyl hydroperoxide; d-GalN, D-galactosamine; HO-1, heme oxygenase-1; Nrf2, nuclear factor erythroid 2-related factor 2; MAPK, mitogen-activated protein kinase; Bax, Bcl-2-associated X protein; Bcl-2, B-cell lymphoma 2; GCLC, glutamate-cystein ligase catalytic subunit; NQO1, NAD(P)H quinone dehydrogenase 1; ATF3, cyclic AMP-dependent transcription factor; and NFκB, nuclear factor kappa B. Upward pointing arrow (↑) and downward pointing arrow (↓) represent an increase and a decrease in gene/protein expression or numerical values, respectively.

As depicted in Figure 2, Hyp serves as an effective hepatoprotective agent by reducing hepatocellular damage due to oxidative stress induced by chemicals [34,35,36,37,40,41,42,43]. Similar antioxidative properties of Hyp were shown in H_2_O_2_-induced intracellular oxidative stress in HepG2 cell [44]. Notably, the regulation of hepatic heme oxygenase-1 (HO-1), nuclear factor erythroid 2-related factor 2 (Nrf2), and mitogen-activated protein kinase (MAPK) in the presence of Hyp may potentiate anti-apoptosis and eventually counteract chemical-driven hepatotoxicity in rodents [34,35,36,37,38,43]. 

The existence of cytochrome P450 2E1 (CYP2E1)-induced hepatotoxicity by cisplatin and acetaminophen is well documented because both drugs undergo hepatic metabolism. CYP2E1, a cytochrome 450 (CYP450) involved in drug-induced liver injury, is predominantly expressed in the liver [45,46]. Although NAC has been considered a standard therapeutic agent for relieving acetaminophen poisoning, there still exist certain limitations for its use due to the possibility of inducing hepatic steatosis and systemic inflammation [47]. Several studies have investigated the efficacy of Hyp in reducing hepatotoxicity in mice and liver cells suffering from drug-induced liver injury [48,49,50,51]. 

These effects of Hyp were associated with the normalization of oxidative stress after high-level administration of acetaminophen or cisplatin with an increase in nuclear Nrf2 levels in the liver [48,49,50,51]. Subsequently, Hyp administration enhanced the hepatic expression of the target genes of Nrf2, such as HO-1—glutamate–cystein ligase catalytic subunit (GCLC)—and NAD(P)H quinone dehydrogenase 1 (NQO1) in LO2 liver cells and C57BL/6 mice in the presence of acetaminophen [49] (Figure 2). However, no study has reported Hyp-induced hepatic steatosis or a systemic inflammatory response similar to that induced by NAC. Hyp isolated from the leaves of *Juglans sinensis* exerted protective effects against hepatotoxicity in HepG2 cells induced by amiodarone and nitrofurantoin; however, the efficacy was not significant [52].

The hepatoprotective efficacy of Hyp has also been evaluated in experimental models of various diseases, including heart failure, pneumonia, hepatitis B, hepatic ischemia, and diabetes [53,54,55,56,57,58,59,60]. Hyp administration resulted in a significant reduction in serum AST and ALT levels and ameliorated the severity of hepatocellular necrosis and vacuolation. In particular, Hyp interfered with ischemia-induced oxidative stress by increasing hepatic proteins HO-1 and NQO1, thus, eventually decreasing apoptotic cells in the liver of hepatic ischemia-reperfusion injury Wistar rats [57]. 

A similar mechanism of Hyp, involved in reducing oxidative stress and inducing anti-apoptosis, was investigated in another study showing hepatotoxicity due to hyperglycemia [53] (Figure 2). Hyp also decreased serum AST and ALT levels by reversing the hepatic malondialdehyde (MDA) and superoxide dismutase (SOD) contents in ApoE^−/−^ mice fed high-fat diet and concanavalin A-induced Kunming mice [61,62]. These studies suggest that Hyp treatment has considerable beneficial effects on hepatotoxicity induced by toxic substances, drugs, and diseases (Table 1). Therefore, further studies are needed to support the efficacy of Hyp and expand its clinical use as an effective hepatoprotectant for the treatment of various liver diseases. 

### 4.2. Antiviral Effects

Over the past decades, viral infection has been regarded as a major risk factor for chronic liver disease [64]. Although higher vaccination coverage and new antivirals have helped decrease the prevalence of hepatitis B virus (HBV) and hepatitis C virus (HCV) cases, viral hepatitis remains a crucial cause of chronic liver diseases [65]. Moreover, the incapability of viral eradication, intolerance, and adverse effects of antiviral agents have been described as limitations in the treatment of viral hepatitis.

Hyp exerts antiviral effects against both HBV and HCV (Table 2). In ducklings inoculated with duck HBV DNA, Hyp at doses of 25–300 mg/kg significantly suppressed serum HBV DNA [54,66,67]. It is noteworthy that Hyp reduced intrahepatic covalently closed circular DNA (cccDNA), serving as a stable template for HBV replication in infected cells, and decreased the secretion of Th1 cytokines [68]. This involvement of Hyp in HBV cccDNA inhibition and the immune response to viral infection may be promising for the development of Hyp as an antiviral. In addition, the viral rebound of HBV in the presence of Hyp in ducklings was lower than that of ducklings treated with lamivudine, a nucleoside reverse transcriptase inhibitor against HBV [54,67]. 

Furthermore, the antiviral effects of Hyp from *Abelmoschus manihot* against the secretion of HBsAg and HBeAg were stronger than lamivudine-induced suppression in HepG2.2.15 cells at the same dose of 0.05 g/kg [54]. Additionally, Hyp inhibited HCV replication by prohibiting HCV NS3 protease via docking the binding sites of NS3 in Huh-7 cells transfected with the NS3 gene of HCV [69]. Thus, further studies are needed to explore the mechanisms underlying the antiviral activities of Hyp to support its function in ameliorating viral hepatitis.

### 4.3. Antisteatotic Effects

The liver is one of the major organs that regulate the fat metabolism [70]. Impairment of hepatic lipid metabolism may induce abnormal lipid accumulation in liver tissue. Hepatic steatosis is an adaptive condition that responds to lipid toxicity; however, various hepatic disorders, including NAFLD, alcoholic liver disease and drug-induced liver injury, often progress from hepatic steatosis [71,72]. In particular, as the prevalence of obesity is sharply increasing, fat-rich diet-induced hepatic steatosis is emerging as a major liver disease [73]. 

Although previous studies on the antisteatotic effects of Hyp are scarce, this compound has been reported to reduce hepatic fat accumulation in rodents fed a high-fat diet and alcohol and in diabetes-induced rats [39,60,61,74,75,76]. The antisteatotic activity of Hyp in high-fat diet-induced C57BL/6 mice and diabetes-induced rats occurred with liver weight loss and reduced hepatic lipid contents, such as TG, TC, and NEFA [60,74]. 

Regarding the underlying mechanisms attributed to the antisteatotic effects of Hyp, it exerted beneficial actions in (1) maintaining hepatic lipid and glucose homeostasis by activating peroxisome proliferator-activated receptor gamma (PPARγ), which lowers glucose levels and reverses lipotoxicity; (2) reducing oxidative stress; (3) synthesizing bile acids from cholesterol by activating key catalytic enzymes CYP7A1 and CYP27A1; (4) increasing the β-oxidation of free fatty acids by the activation of nuclear farnesoid X receptor (FXR) and transcription factor liver X receptor (LXR)α implicated in lipid oxidation; and (5) inhibiting de novo lipogenesis by modulating hepatic de novo lipogenesis markers acetyl-CoA carboxylase (ACC) and sterol regulatory element binding proteins (SREBP)1,2 in vivo and in vitro [39,61,74,75,76] (Table 3). 

### 4.4. Anti-Inflammatory Effects

The liver has recently been regarded as an important immunologic and metabolic organ [77]. Kupffer cells, innate lymphocytes, and many antigen-presenting cells are enriched in the liver tissue, and their unique immune microenvironment is closely associated with inflammatory reactions in the liver [78]. In particular, inflammatory processes are inevitably involved in the development of various liver diseases, including viral infections, autoimmune hepatitis, alcoholic hepatitis, and nonalcoholic steatohepatitis (NASH) [78]. Thus, therapeutic strategies that interfere with inflammatory markers, such as anti-tumor necrosis factor (TNF)-α treatment (e.g., infliximab and etanercept) and interleukin-24 therapy, are being investigated for the treatment of liver diseases [79,80]. 

The hepatic expression of several key inflammatory cytokines and chemokines, such as TNF-α, interleukin (IL)-1β, IL-6, nitric oxide (NO), inducible nitric oxide synthase (iNOS), cyclooxygenase (COX)2, C-C motif chemokine ligand (CCL)2, and CCL5, was downregulated by the administration of Hyp during the inflammatory pathogenesis induced by CCl_4_ and a high-fat diet [34,38,53,60,76].

The neutralization of inflammatory markers by Hyp in hepatic tissue was accompanied by marked improvement in histological findings from abnormal conditions, including hepatic inflammatory cell infiltration and Kupffer cell hyperplasia [34,38,53,76]. Hyp obtained from *Artemisia capillaris* and *Zanthoxylum bungeanum* leaves displayed anti-inflammatory effects against CCl_4_ and high-carbohydrate/high-fat diet and alloxan-induced inflammation via hepatic removal of oxidative stress, respectively [34,53]. 

Hyp also lowered hepatic inflammatory cell infiltrations through the amelioration of hepatic oxidative stress induced by concanavalin A, high-fat diet, and alcohol [39,61,62]. In addition, the regulation of other MAPKs, NFκB, and apoptotic factors also suggests that Hyp controls inflammation [38,53,60] (Table 4). In this respect, antioxidant, anti-apoptosis, and the regulation of MAPKs and NFκB may be mechanisms that support Hyp as a beneficial anti-inflammatory agent against hepatic inflammation. 

### 4.5. Antifibrotic Effects

Liver fibrosis is a vital process in wound healing that occurs during liver injury; however, it is associated with the deformation of normal hepatocytes, collagen deposition, and overaccumulation of the extracellular matrix (ECM) [81]. Fibrosis reaction and severity largely influence the overall prognosis, exacerbation, and management of CLD because liver stiffness is a significant condition that increases the risk of liver-related disorders and all-cause deaths [82,83,84]. Hence, blocking fibrogenesis pathways may play a crucial role in managing patients with CLD.

Hepatic stellate cell (HSC) activation is a common process in the pathogenesis of liver fibrosis, which subsequently triggers the excessive deposition of ECM in liver tissue. Regulation of HSC proliferation and activation is suggested to be a key therapeutic strategy that prevents the progression of hepatic fibrosis in different liver disorders. Hyp inhibits the proliferation of LX-2 human HSC line via apoptosis induction and intracellular ROS reduction [85]. The inhibition of LX-2 cells by Hyp was found to induce significant downregulation of α-smooth muscle actin (α-SMA) and collagen I mRNA and protein expression, which increased during HSC activation [85]. 

Similar results were observed in the presence of Hyp in transforming growth factor (TGF)-β-induced LX-2 cells [55]. TGF-β signaling is often referred to as an important pathway involved in different stages of liver disease progression, and high levels of TGF-β may be a cause and consequence of liver damage [86]. With regard to TGF-β overactivation in hepatic tissue induced by a high-fat diet and heart failure, Hyp significantly inhibited hepatic TGF-β expression. The inactivation of TGF-β signaling by Hyp may contribute to the prevention of liver cirrhosis and cancer initiation [86]. 

In addition to targeting TGF-β, Hyp improved histological findings exhibiting a hepatic fibrotic area increased by different etiologies, such as CCl_4_, a high-fat diet, and heart failure in mice and rats [35,55,76]. In particular, Hyp ameliorated CCl_4_-induced liver fibrosis and heart failure-induced hepatic fibrosis, accompanied by hepatocellular antioxidant defenses [35,59]. Since Hyp lowered the ROS levels in LX-2 cells, and various antioxidants are currently used to treat liver fibrosis, the antifibrotic effects of Hyp via targeting oxidative stress should be explored to counteract liver fibrosis (Table 5).

### 4.6. Anticancer Effects

Hepatocellular carcinoma (HCC) is a predominant tumor type that develops from hepatocytes and is a major cause of cancer-related mortalities [87]. Due to late diagnosis, frequent recurrence, and impaired liver function, HCC has a poor prognosis with high mortality and a low therapeutic rate [88]. In particular, the HCC disease burden is considerable in areas with a high prevalence of HBV and HCV infection because the majority of HCC cases are attributed to chronic hepatitis B and C [89,90]. 

Moreover, the number of patients with NASH-related HCC is steadily increasing in developed countries. However, the therapeutic efficacy of current chemotherapeutic agents remains limited, and recurrence after HCC treatment often occurs. Hence, preemptive management and modification of HCC-predisposing factors to reduce HCC development may serve as an effective option. Furthermore, complementary therapy to alleviate the adverse effects of anticancer drugs and enhance their efficacy is needed.

First, Hyp significantly suppressed the survival rates of both HepG2 and insulin-resistant HepG2 cells [75,91,92]. In particular, the cytotoxic effects of Hyp against HepG2 cells may involve cell cycle arrest at G0/G1 through the downregulation of cyclin-D1 and c-Myc expression, and apoptosis induction via the activation of p53/caspase pathway [91]. Moreover, Hyp attenuates the expression of bone morphogenetic protein (BMP)-7, p-protein kinase B (AKT), and phosphoinositide 3-kinase (PI3K), which are involved in inducing metastasis, along with the antiproliferation of HCC [91]. 

Second, Hyp may be a promising compound against HBV-related HCC. PLC-PRF-5 cells, the Alexander hepatoma cell line, grow continuously and secrete HBV surface antigens [93]. Hyp significantly inhibited HCC growth and increased the average survival rates of BALB/c mice injected with PLC-PRF-5 cells [94]. More importantly, Hyp treatment decreased tumor migration and invasion in PLC-PRF-5 high metastatic cells, and this antimetastatic effect of Hyp was associated with the regulation of various factors involved in the epithelial–mesenchymal transition (EMT) by increasing hepatic E-cadherin and suppressing the expression of quaking, circRNAs, and hepatic vimentin [94]. Eventually, Hyp downsized metastatic lung nodules in an HCC in vivo model [94]. Hence, Hyp may affect metastatic properties, such as migration, the invasion of other organs, the promotion of HCC EMT, and tumor growth (Table 6).

## 5. Safety of Hyperoside

According to recent studies, a few studies have investigated the safety of Hyp [14,95]. In 40 healthy BALB/c mice, Hyp treatment (5,000 mg/kg, intragastrical route once) did not result in mouse poisoning, death, acute toxicity, or genotoxicity [96]. In addition to the toxicity evaluation after administration of high-dose Hyp in mice, Wistar rats were orally administered with Hyp (30, 175, and 1000 mg/kg) for 6 months to investigate the long-term toxicity of Hyp [97]. No significant changes in rat behavior, food-intake, and weight gain were observed in the presence of chronic treatment of Hyp by gavage. However, renal interstitial inflammatory cell infiltration was observed in about 20% in 20 rats of the high-dose (1000 mg/kg) group of Hyp. 

At the end of the recovery period of 1 month, the renal pathological injuries were gradually diminished. Although Hyp may induce renal toxicity, this can be reversible after withdrawal of the compound. In addition, Hyp treatment (8, 65, and 500 mg/kg) for 38 weeks induced no abnormal reactions in symptoms, blood tests, and histological examination in Beagle dogs, and mild toxicity of the kidney and liver was negligible and reversible [98]. Other teams found that Hyp can contribute to the alleviation of renal cellular aging and injury in NRK-52E cells and rats exposed to D-galactose [99] as well as the protection of HK-2 (human renal proximal tubule cells) from high-glucose-induced apoptosis and inflammation [100]. 

Thus, it is still controversial whether Hyp induces renal and hepatic toxicity, and further studies to decide the appropriate dosages and treatment periods applicable to clinical use are needed, particularly for patients with renal or hepatic dysfunction. In terms of the impact of Hyp on fetal mice, 30 and 175 mg/kg Hyp treatment showed no significant changes in fetal appearance and growth; however, high-dose Hyp (1000 mg/kg) administration during 6–15 days after gestation significantly retarded the growth of fetus (weight and the lengths of embryo and tail) of pregnant Wistar rats [101]. 

On the other hand, a recent study reported that Hyp (40 mg/kg) increased the body weight of fetuses and decreased pregnancy loss in a rat model with recurrent pregnancy loss [102]. Eventually, a proper administration of Hyp based on a safe dosage setting may ensure that pregnant women can safely receive treatment depending on their indications. Consequently, although Hyp, a dietary flavonoid, showed strong antioxidant and hepatoprotective potency, further evaluation of the safety of Hyp needs to be performed due to the lack of evidence in experimental models.

## 6. Discussion

**Figure 3:**
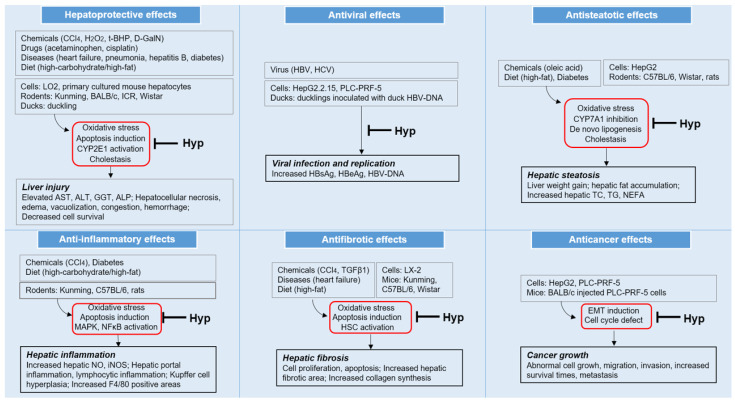
Pharmacological effects and underlying mechanisms of hyperoside (Hyp) in different pathological conditions related to various liver diseases; CCl_4_, carbon tetrachloride; H_2_O_2_, hydrogen peroxide; t-BHP, tert-butyl hydroperoxide; d-GalN, D-galactosamine; CYP, cytochrome P450; AST, aspartate aminotransferase; ALT, alanine aminotransferase; GGT, gamma glutamyl peptidase; ALP, alkaline phosphatase; TC, total cholesterol; TG, triglyceride; NEFA, nonesterified fatty acids; MAPK, mitogen-activated protein kinase; NFκB, nuclear factor kappa-light-chain-enhancer of activated B cells; NO, nitric oxide; iNOS, inducible nitric oxide synthase; HSC, hepatic stellate cell; and EMT, epithelial–mesenchymal transition.

This review summarizes the beneficial activities of Hyp associated with liver disease and the underlying signaling mechanisms in vitro and in vivo reported in experimental studies. As illustrated in Figure 3, Hyp has been reported to exert hepatoprotective, antiviral, antisteatotic, anti-inflammatory, antifibrotic, and anticancer effects. Hyp displayed its pharmacological effects by regulating multiple mechanisms, including: (1) the depletion of hepatocellular oxidative state through the modulation of Nrf2 and ATF3; (2) the regulation of several anti-apoptotic factors; (3) the activation of hepatic metabolism on cholestasis and lipogenesis; (4) the suppression of HSC activation; and (5) the inhibition of EMT induction and the induction of cell cycle arrest (Figure 3).

For most liver pathologies, ROS are involved in different reactions that generate hepatic steatosis, inflammation, and fibrosis, and an impaired redox system in hepatic tissue can cause direct/indirect hepatic injury. Thus, enhancement of the antioxidant defense system by Hyp, consisting of reduced ROS generation and increased ROS elimination in hepatic tissue, may be one of the major molecular mechanisms of Hyp. In particular, major sources of oxidative stress involved in the therapeutic targets of Hyp are derived from CYP2E1 and the downregulation of the expression of antioxidant genes and Nrf2-regulated phase II enzymes occurring in liver disorders.

**Figure 4:**
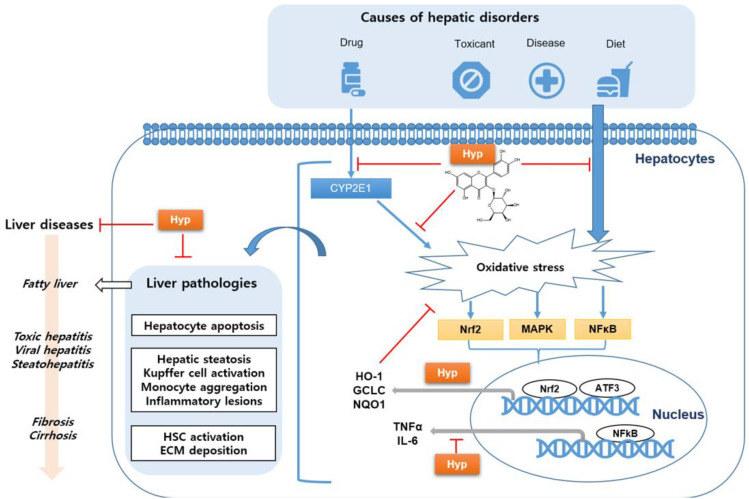
Schematic diagram of hyperoside (Hyp), which improves various liver diseases by reducing oxidative stress; CYP, cytochrome P450; Nrf2, nuclear factor erythroid 2-related factor 2; MAPK, mitogen-activated protein kinase; NFκB, nuclear factor kappa-light-chain-enhancer of activated B cells; HO-1, heme oxygenase-1; GCLC, glutamate–cystein ligase catalytic subunit; NQO1, NAD(P)H quinone dehydrogenase 1; TNF-α, tumor necrosis factor-alpha; IL-6, interleukin-6; ATF3, cyclic AMP-dependent transcription factor; HSC, hepatic stellate cell; and ECM, extracellular matrix.

As depicted in Figure 4, changes in the redox and detoxifying potential of hepatocytes induced by drugs, toxic substances, diseases, and a high-fat diet can result in oxidative-stress-derived liver injury, as demonstrated by the decreased formation of hepatic antioxidant enzymes and high levels of ROS in the liver. This liver damage related to oxidative stress is represented by liver pathologies, such as hepatocyte apoptosis, hepatic steatosis, Kupffer cell activation, monocyte aggregation, HSC activation, and ECM deposition. Thus, the imbalance between antioxidant and pro-oxidant conditions can induce progressive liver diseases, including simple fatty liver, NASH, toxic hepatitis, viral hepatitis, and liver cirrhosis. 

Hyp has been demonstrated to exert marked scavenging activities against ROS attack occurring in hepatocytes induced by a variety of liver diseases. Particularly, Hyp displayed beneficial activity against CYP2E1-dependent oxidative stress and hepatotoxicity induced by drugs, such as paracetamol and cisplatin, which activated the upregulation of Nrf2 and the induction of Nrf2-targeted genes for detoxification in hepatic cells. Additionally, Hyp regulated the oxidative-stress-induced alterations of MAPK and NFκB as well as Nrf2, which impact transcription factors, thereby, resulting in the production of detoxifying enzymes and inflammatory cytokines. 

Eventually, Hyp showed biological actions in experimental models against oxidative stress that disrupt the physiological liver function or normal architectural structure of liver. These antioxidant actions of Hyp may be closely associated with its chemical structure, particularly depending on the main structure and position of the galactose sugar moiety attached to this compound. For instance, Hyp has been reported to exhibit excellent antioxidant potency in in vitro and in vivo studies, and its activity was influenced by the presence of galactose at the position C3 and β-hydroxyl groups at C4 and C6 as well as the antioxidant structure–activity relationship (SAR) [44,60]. 

In addition, the hydroxyl groups in positions 3’ and 4’ of ring B of Hyp were found to possess high *OH-scavenging activity [103]. Hence, further investigations on SAR analysis of Hyp need to be performed to elucidate the SAR relationship of beneficial actions of Hyp against liver diseases.

In addition to the Hyp-induced alterations of oxidative stress against liver injury, it has been reported that several mechanisms are involved in the pharmacological effects of Hyp responsible for ameliorating viral infection, steatosis, and HCC. Hyp exerted antiviral effects via the inhibition of the formation of hepatic cccDNA along with the suppressed Th1 cytokine secretion in mouse spleen lymphocytes. In regard to hepatic steatosis, Hyp attenuated the lipid accumulation in liver by preserving hepatic lipid and bile acid homeostasis via the regulation of PPARγ, FXR, LXRα, ACC, SREBP, cholesterol 7α-hydroxylase (CYP7A1), and CYP27A1. It has also been demonstrated that the anticancer properties of Hyp are attributed to cell cycle arrest and the PI3K/AKT signaling pathway.

Since the early 1960s, Hyp has been reported to be isolated from over 30 herbal plants via various extraction technologies; however, Hyp involved in the effects for the treatment of liver diseases in the present study has been isolated from eight species of medicinal plants: Hyp derived from *Artemisia capillaris* and *Zanthoxylum bungeanum* showed hepatoprotective and anti-inflammatory effects; Hyp obtained from *Abelmoschus manihot* exerted hepatoprotective and antiviral effects; Hyp from *Zanthoxylum schinifolium*, *Apocynum venetum*, *Canarium album*, and *Cuphobia nematocypha* showed hepatoprotective activities; and Hyp from *Hypericum patulum* had antisteatotic effects. 

Among them, further investigation is required to evaluate the role of Hyp as an active constituents of *Artemisia capillaris*—a well-known herbal plant frequently used for treating liver disease [104]. In addition, based on above medicinal plants containing Hyp, further studies on efficient and economic extraction methods maximizing the purity and yield are needed [14,95]. Hence, Hyp may be an active compound present in various herbal plants that can be widely applied in clinical settings owing to its hepatoprotective, antiviral, antisteatotic, anti-inflammatory, antifibrotic, and anticancer activities. 

In addition, the antioxidant properties of Hyp can be extended to the suppression of glycative stress, which can take place in various liver diseases [105]. Although Hyp attenuated advanced glycation end-products (AGEs) in ECV304 bladder cancer cells and suppressed apoptosis and necrosis in AGEs-induced podocytes, there still exists no study describing the relationship of antiglycant effects of Hyp with liver disorders [106,107]. Thus, relevant further studies need to be performed to strengthen and support pharmacological effects of Hyp in liver disorders.

This review provides evidence for the use of Hyp in various liver diseases. Most liver diseases, such as NAFLD, DILI, hepatitis, and liver cirrhosis, are closely associated with oxidative-stress-induced injury, particularly in the early stages of hepatic disorders. Hence, Hyp can improve liver function and reverse pathological changes, which are mainly mediated by antioxidant signaling.

## 7. Conclusions

Hyp can serve as a potential therapeutic agent against various hepatic pathological changes, including liver injury, steatosis, viral infection, inflammation, fibrosis, and cancer, mainly by reducing oxidative stress, inhibiting apoptosis, and regulating the lipid and cholesterol metabolism. In particular, the present review highlights the multipotent antioxidant mechanism of Hyp, and these antioxidant properties can be useful in mitigating hepatic disorders but not for cancer. Therefore, Hyp can be considered to be a beneficial compound that exhibits a wide range of biological actions with minimal hepatotoxicity in various liver diseases.

## Figures and Tables

**Table 1:** Hepatoprotective activities of hyperoside (Hyp) in experimental models related to various liver diseases.

Hyperoside/Source	Experimental Model	Dose	Results/Molecular Mechanisms	References
Hyp/*Artemisia* *capillaris*	CCl_4_-induced liver injury in mice	50, 100, 200 mg/kg	↓serum AST, ALT ↓centrizonal necrosis ↓hepatic MDA ↑hepatic GSH ↑hepatic Nrf2 protein ↑hepatic HO-1 mRNA and protein	[34]
Hyp	CCl_4_-induced male Kunming mice	200, 400 mg/kg	↓serum AST, ALT ↓hepatocellular necrosis ↓hepatic MDA, SOD, GSH-Px ↑hepatic CAT ↑hepatic Nrf2	[35]
Hyp	CCl_4_-induced male Sprague-Dawley rats	60 mg/kg	↓serum AST, ALT ↓liver cell edema, nuclear condensation, hepatocellular vacuolization ↓hepatic MDA ↑hepatic SOD ↑hepatic p-AKT, p-GSK-3β ↑hepatic nuclear Nrf2 ↑hepatic HO-1 ↓hepatic p-Fyn ↓hepatic PHLPP2	[36]
Hyp	CCl_4_-induced male BALB/c mice	50, 100 mg/kg	↓serum AST, ALT ↓hepatocellular vacuolization ↓hepatic MDA, SOD ↑hepatic GSH	[37]
Hyp	CCl_4_-induced C57BL/6J mice	200 mg/kg	↓serum AST, ALT ↓hepatocyte destruction ↓hepatic p-p38, p-Erk protein	[38]
Hyp	CCl_4_-induced rats	30, 60 mg/kg	↓hepatic focal necrosis ↓hepatic MDA ↑hepatic SOD, GSH	[40]
Hyp/*Zanthoxylum schinifolium*	CCl_4_-induced mice	10, 20 mg/kg	↓serum AST, ALT ↓hepatic TBARS	[41]
Hyp/*Canarium album* and *Euphorbia* *nematocypha*	CCl_4_-induced primary cultured rat hepatocytes	1, 10 µM	↓MDA	[42]
Hyp/*Zanthoxylum bungeanum* leaves	High-carbohydrate/high-fat diet and alloxan-induced male Kunming mice	200 mg/kg	↓hepatic AST, ALT ↑hepatic Na+/K+ ATPase ↓hepatic focal necrosis ↑hepatic SOD, GSH, CAT ↓hepatic MDA ↓hepatic ATF3 ↓hepatic p-p65 ↓hepatic p-p38, p-Erk1/2, p-JNK ↓hepatic Bax ↑hepatic Bcl-2 ↓hepatic caspase 3,9 ↓hepatic cytochrome c	[53]
Hyp	ApoE^−/−^ mice fed high-fat diet	200 mg/kg	↓serum AST, ALT ↓hepatic MDA ↑hepatic SOD, GSH-Px	[61]
Hyp	Kunming mice given 50% alcohol	25, 50 mg/kg	↓serum AST, ALT ↓hepatocellular necrosis and edema ↓hepatic MDA ↑hepatic SOD, GSH	[39]
Hyp/*Abelmoschus manihot*	Ducklings inoculated with HBV-DNA	0.1 g/kg/day	↓hepatocellular necrosis ↓hepatocellular vacuolation	[54]
Hyp	Ducklings inoculated with duck HBV DNA	60 mg/kg	↓hepatic ALT ↓hepatic cord derangement	[58]
Hyp	Heart failure-induced liver fibrosis in male Wistar rats	100, 200 mg/kg	↓serum AST, ALT ↓serum ALP	[55]
Hyp	Heart failure-induced liver fibrosis in male Wistar rats	100, 200 mg/kg	↓serum AST, ALT ↓hepatic MDA ↑hepatic SOD, GSH-Px	[59]
Hyp	Diabetes-induced rats	10 mg/kg	↓serum AST, ALT	[60]
Hyp	Pneumonia-induced liver injury in BALB/c mice	12.5, 50 mg/kg	↓serum AST, ALT	[56]
Hyp	Cisplatin-induced liver injury in male ICR mice	50 mg/kg	↓serum AST, ALT, GGT ↓hepatocellular vacuolation ↓hepatic MDA ↑hepatic T-AOC, SOD, CAT, GSH, GSH-Px, GST	[48]
Hyp	H_2_O_2_-induced LO2 liver cells	100 µM	↑cell survival rate, ↓LDH leakage ↑GSH ↑HO-1 mRNA and protein ↑nuclear Nrf-2 mRNA and protein ↑ARE, p-GSK-3β	[43]
Hyp	H_2_O_2_-induced LO2 liver cells	100, 200 µM	↓MDA, ROS ↑HO-1 ↑ARE ↑nuclear Nrf-2 ↓nuclear Bach1 ↑Crm1 ↑Erk1/2	[37]
Hyp	H_2_O_2_-induced HepG2 cells	1, 10 µM	↓ROS	[44]
Hyp	t-BHP-induced LO2 liver cells	100 µM	↑HO-1 ↑nuclear Nrf-2 ↓p-Fyn ↑p- GSK-3β ↑p-Akt ↓PHLPP2	[36]
Hyp	Concanavalin A-induced Kunming mice	25, 50 mg/kg	↓serum AST, ALT ↓hepatocellular necrosis ↓hepatic MDA ↑hepatic SOD	[62]
Hyp/*Apocynum venetum*	D-GalN/TNF-α-induced primary cultured mouse hepatocytes	20, 40, 80 µM	↑cell survival rate	[63]
Hyp/*Canarium album* and *Euphorbia nematocypha*	D-GalN-induced primary cultured rat hepatocytes	3, 10, 30 µM	↓ALT	[42]
Hyp	Acetaminophen-induced LO2 liver cells	10, 20 µM	↑cell survival rate ↓LDH ↓ALT ↑nuclear Nrf2 ↑HO-1, GCLC, NQO1	[49]
Hyp	Acetaminophen-induced male Kunming mice	100 mg/kg	↓serum AST, ALT ↓liver congestion, centrilobular necrosis ↑hepatic UGTs ↑hepatic SULTs ↓hepatic CYP2E1 ↑nuclear Nrf-2 mRNA and protein	[50]
Hyp	Acetaminophen-induced male C57BL/6 mice	25, 50, 100 mg/kg	↓serum AST, ALT, ALP ↓hepatic MDA ↑hepatic GSH, SOD, GST, GSH-Px ↑hepatic nuclear Nrf2 ↑hepatic HO-1, GCLC, NQO1	[49]
Hyp	Acetaminophen-induced male C57BL/6 mice	60 mg/kg	↓serum AST, ALT ↓hepatocellular vacuolation, lintrahepatic hemorrhage, lymphocyte infiltration ↓hepatic ROS, MDA ↑hepatic GSH, GST, GSH-Px ↓hepatic CYP2E1 mRNA and protein	[51]
Hyp	Hepatic ischemia-reperfusion injury male Wistar rats	50 mg/kg	↓serum AST, ALT ↓Suzuki score ↓hepatic MDA ↑hepatic SOD, GSH-Px ↑hepatic HO-1, NQO1 protein ↓apoptotic cells in liver ↑hepatic Bcl-2 protein ↓hepatic Bax, caspase-3 protein	[57]

CCl_4_, carbon tetrachloride; AST, aspartate aminotransferase; ALT, alanine aminotransferase; MDA, malondialdehyde; GSH, glutathione; Nrf2, nuclear factor erythroid 2-related factor 2; HO-1, heme oxygenase-1; SOD, superoxide dismutase; GSH-Px, glutathione peroxidase; CAT, catalase; AKT, protein kinase B; GSK-3β, glycogen synthase kinase-3β; PHLPP2, PH domain and leucine rich repeat protein phosphatase 2; Erk, extracellular signal-regulated kinase; TBARS, thiobarbituric acid reactive substance; ATF3, cyclic AMP-dependent transcription factor; JNK, c-Jun N-terminal kinase; Bax, Bcl-2-associated X protein; Bcl-2, B-cell lymphoma 2; ALP, alkaline phosphatase; GGT, gamma glutamyl peptidase; LDH, lactate dehydrogenase; ARE, antioxidant response element; Bach1, BTB domain and CNC homolog 1; Crm1, chromosome region maintenance 1; NQO1, NAD(P)H quinone dehydrogenase 1; UGTs, UDP-glucuronosyltransferases; SULTs, sulfotransferases; and CYP, cytochrome P450. Upward pointing arrow (↑) and downward pointing arrow (↓) represent an increase and a decrease in gene/protein expression or numerical values, respectively.

**Table 2:** Antiviral activities of hyperoside (Hyp) in experimental models related to hepatitis B virus (HBV) and hepatitis C virus (HCV) infection.

Hyperoside/Source	Experimental Model	Dose	Results/Molecular Mechanisms	References
Hyp/*Abelmoschus manihot*	HepG2.2.15 cells	0.0125, 0.025, 0.05 g/L	↓HBsAg ↓HBeAg	[54]
Hyp/*Abelmoschus manihot*	Ducklings inoculated with duck HBV DNA	0.1 g/kg/day	↓serum HBV DNA	[54]
Hyp	Duck HBV infection model and normal mouse spleen lymphocyte	25, 50 mg/kg	↓serum HBV DNA ↓hepatic cccDNA ↓Th1 cytokine in normal mouse spleen lymphocyte	[66]
Hyp	Ducklings inoculated with duck HBV DNA	300 mg/kg	↓serum HBV DNA ↓rebound of serum HBV DNA compared with lamivudine	[67]
Hyp	Huh-7 cells transfected with NS3 gene of HCV	Not known	↓HCV NS3 protease by docking the binding sites of NS3 protein	[69]

HBV, hepatitis B virus; HCV, hepatitis C virus; NS3, nonstructural protein. Upward pointing arrow (↑) and downward pointing arrow (↓) represent an increase and a decrease in gene/protein expression or numerical values, respectively.

**Table 3:** Antisteatotic activities of hyperoside (Hyp) in experimental models related to hepatic steatosis.

Hyperoside/Source	Experimental Model	Dose	Results/Molecular Mechanisms	References
Hyp	High-fat diet-induced male C57BL/6 mice	50 mg/kg	↓liver weight ↓hepatic fat accumulation ↓hepatic TG, TC, NEFA	[74]
Hyp	Diabetes-induced rats	10 mg/kg	↓liver weight ↓hepatic TG, TC ↓hepatic steatosis score	[60]
Hyp/*Hypericum patulum*	Oleic acid-treated HepG2 cells	2.5, 5 µM	↓fat accumulation ↓TG contents ↓ROS ↑PPARγ	[75]
Hyp	ApoE^-/-^ mice fed high-fat diet	200 mg/kg	↓hepatic fat accumulation ↓hepatic MDA ↑hepatic SOD, GSH-Px	[61]
Hyp	Wistar male rats fed high-fat diet	0.6, 1.5 mg/kg	↓hepatic fat accumulation ↑hepatic CYP7A1, CYP27A1 ↑hepatic FXR, LXRα ↑hepatic ACC, pACC ↓hepatic SREBP1,2	[76]
Hyp	Kunming mice given 50% alcohol	25, 50 mg/kg	↓ hepatic fat accumulation ↓hepatic MDA ↑hepatic SOD, GSH	[39]

TG, triglyceride; TC, total cholesterol; NEFA, non-esterified fatty acids; ROS, reactive oxygen species; PPARγ, peroxisome proliferator- activated receptor gamma; CYP, cytochrome P450; FXR, farnesoid X receptor; LXR, liver X receptor; ACC, acetyl-CoA carboxylase; and SREBP, Sterol regulatory element binding proteins. Upward pointing arrow (↑) and downward pointing arrow (↓) represent an increase and a decrease in gene/protein expression or numerical values, respectively.

**Table 4:** Anti-inflammatory activities of hyperoside (Hyp) in experimental models related to hepatic inflammation.

Hyperoside/Source	Experimental Model	Dose	Results/Molecular Mechanisms	References
Hyp/*Artemisia* *capillaris*	CCl_4_-induced liver injury in mice	50, 100, 200 mg/kg	↓Portal inflammation ↓Kupffer cell hyperplasia ↓hepatic iNOS, COX2 mRNA and protein ↑hepatic HO-1 mRNA and protein ↑Nrf2 protein	[34]
Hyp	CCl_4_-induced C57BL/6J mice	200 mg/kg	↓hepatic TNF-α, IL-6 protein ↓hepatic inflammatory cells infiltrations ↓hepatic p-p38, p-Erk protein	[38]
Hyp	Concanavalin A-induced Kunming mice	25, 50 mg/kg	↓hepatic inflammatory cells infiltrations ↓hepatic MDA ↑hepatic SOD	[62]
Hyp/*Zanthoxylum bungeanum* leaves	High-carbohydrate/high-fat diet and alloxan-induced male Kunming mice	200 mg/kg	↓hepatic NO, iNOS ↓lymphocytic inflammation ↑hepatic SOD, GSH, CAT ↓hepatic MDA ↓hepatic p-p65 ↓hepatic p-p38, p-Erk1/2, p-JNK ↑hepatic Bcl-2 ↓hepatic Bax, caspase-3,9, cytochrome c	[53]
Hyp	ApoE^-/-^ mice fed high-fat diet	200 mg/kg	↓hepatic inflammatory cells infiltrations ↓hepatic MDA ↑hepatic SOD, GSH-Px	[61]
Hyp	High-fat diet-induced male C57BL/6 mice	50 mg/kg	↓hepatic F4/80 positive areas ↓hepatic TNF-α, IL-1β, IL-6, CCL2, CCL5, iNOS mRNA	[74]
Hyp	Diabetes-induced rats	10 mg/kg	↓hepatic TNF-α protein ↓hepatic NFκB protein ↓hepatic inflammation score	[60]
Hyp	Kunming mice given 50% alcohol	25, 50 mg/kg	↓hepatic inflammatory cells infiltrations ↓hepatic MDA ↑hepatic SOD, GSH	[39]

CCl_4_, carbon tetrachloride; phosphatase 2; TNF-α, tumor necrosis factor-alpha; iNOS, inducible nitric oxide synthase; COX2, cyclooxygenase2; HO-1, heme oxygenase-1; Nrf2, nuclear factor erythroid 2-related factor 2; IL-6, interleukin-6; Erk, extracellular signal-regulated kinase; NO, nitric oxide; SOD, superoxide dismutase; GSH, glutathione; CAT, catalase; MDA, malondialdehyde; JNK, c-Jun N-terminal kinase; Bcl-2, B-cell lymphoma; Bax, Bcl-2-associated X protein; CCL, C-C motif chemokine ligand; and NFκB, nuclear factor kappa-light-chain-enhancer of activated B cells. Upward pointing arrow (↑) and downward pointing arrow (↓) represent an increase and a decrease in gene/protein expression or numerical values, respectively.

**Table 5:** Antifibrotic activities of hyperoside (Hyp) in experimental models related to hepatic fibrosis.

Hyperoside/Source	Experimental Model	Dose	Results/Molecular Mechanisms	References
Hyp	LX-2 cells	2 mM/L	↓cell proliferation ↑cell apoptosis rate ↑proapoptotic genes (Bcl-X_s_, DR4, Fas, FasL) ↓antiapoptotic genes (A20, c-IAP1, Bcl-X_L_, RIP1) ↓α-SMA, collagen I mRNA and protein ↓intracellular ROS ↓TNF-α-induced NFκB p65 DNA binding (by Hyp 1mM/L)	[85]
Hyp	CCl_4_-induced male Kunming mice	200, 400 mg/kg	↓serum MAO ↓hepatic MAO ↓fibrosis around central vein ↓hepatic MDA ↑hepatic SOD, GSH-Px, CAT ↑hepatic Nrf2	[35]
Hyp	High-fat diet-induced male C57BL/6 mice	50 mg/kg	↓hepatic fibrotic area ↓hepatic Col1A1, CTGF, TGF-β mRNA	[74]
Hyp	Heart failure-induced liver fibrosis in male Wistar rats	100, 200 mg/kg	↓hepatic hydroxyproline ↓hepatic fibrosis area ↓hepatic α-SMA, collagen I, CTGF mRNA and protein ↓hepatic MMP2, MMP9 mRNA and protein ↓hepatic TGFβ1, p-Smad 2,3 protein	[55]
Hyp	Heart failure-induced liver fibrosis in male Wistar rats	200 mg/kg	↓hepatic hydroxyproline ↓hepatic TGF-β1, CTGF, TIMP1, MMP1, MMP2, collagen III mRNA and protein ↓hepatic MDA ↑hepatic SOD, GSH-Px	[59]
Hyp	TGF-β1-induced LX-2 cells	2 mM	↓α-SMA mRNA and protein ↓collagen I mRNA and protein ↓p-Smad 2,3 protein	[55]

Bcl-2, B-cell lymphoma; DR, death receptor; FasL, Fas ligand; c-IAP1, cellular inhibitor of apoptosis protein1; RIP1, receptor interacting protein; α-SMA, α-smooth muscle actin; TNF-α, tumor necrosis factor-alpha; NFκB, nuclear factor kappa-light-chain-enhancer of activated B cells; CCl_4_, carbon tetrachloride; MAO, monoamine oxidase; MDA, malondialdehyde; SOD, superoxide dismutase; GSH-Px, glutathione peroxidase; CAT, catalase; Col1A1, collagen type 1 alpha 1; CTGF, connective tissue growth factor; TGF-β, transforming growth factor-beta; and MMP, matrix metalloproteinase. Upward pointing arrow (↑) and downward pointing arrow (↓) represent an increase and a decrease in gene/protein expression or numerical values, respectively.

**Table 6:** Anticancer activities of hyperoside (Hyp) in experimental models related to hepatocellular carcinoma (HCC).

Hyperoside/Source	Experimental Model	Dose	Results/Molecular Mechanisms	References
Hyp	PLC-PRF-5 hepatoma cells	20, 50 µM	↓cell migration ↓cell invasion ↓quaking ↓circRNAs	[94]
Hyp	BALB/c mice injected with PLC-PRF-5 cells	50, 100 mg/kg	↓tumor growth ↑survival times ↓metastatic lung nodules ↓hepatic quaking ↓hepatic vimentin ↑hepatic E-cadherin	[94]
Hyp	Insulin-resistant HepG2 cells	10 µM	↓cell survival rate	[75]
Hyp	HepG2 cells	10, 20, 40, 80 µM	↓cell survival rate ↓BMP-7 mRNA and protein ↓cyclin-D1 and c-Myc ↑G0/G1 arrest ↓p-AKT, PI3K	[91]
Hyp	HepG2 cells	20, 50 nmol/L	↓cell survival rate ↑nuclear shrinkage ↑cell apoptosis rate ↑p53 protein ↑hepatic caspase-3,9 protein	[92]

BMP-7, bone morphogenetic protein-7; AKT, protein kinase B; and PI3K, phosphoinositide 3-kinase. Upward pointing arrow (↑) and downward pointing arrow (↓) represent an increase and a decrease in gene/protein expression or numerical values, respectively.

## Data Availability

Not applicable.
